# Supplementary material for: Defunctionalizing intracellular organelles such as mitochondria and peroxisomes with engineered phospholipase A/acyltransferases
Source: Nat Commun. 2022 Jul 29;13:4413. doi: 10.1038/s41467-022-31946-5 (PMC9338259; doi:10.1038/s41467-022-31946-5)
Supplement: Supplementary file 1 — Supplementary Information [file 41467_2022_31946_MOESM1_ESM.pdf]

Supplementary Note 1

Spontaneous and stress-induced translocation of PLAAT3

In the cell viability assay, we became aware that FL and FL-LD basically exist in soluble forms but also exhibited local accumulation in most of cells (**Fig. 1e**, insets). In contrast, expression of 18TM did not elicit these punctate signals, suggesting that CT domain was responsible for inducing this punctuation. Though it has been reported that membrane-damage reagents induced murine PLAAT3 translocation onto organellar membranes[1], our result suggested that PLAAT3 may be spontaneously relocalized to organellar membrane by cellular stress as well. Thus, we further examine the property of damage-dependent translocation of 18TM. At first, we used l-Leucyl-l-Leucine methyl ester (LLOME) which was used as a positive control to directly destabilize organellar membrane[1]. After COS-7 cells were transfected with plasmids expressing YFP, YFP-FL, or YFP-18TM (or their mCherry versions) and CFP-MoA, we observed translocating to mitochondria after treatment with 1mM of LLOME or other cellular stress agents. LLOME induced co-localization of mCherry-FL and CFP signals on mitochondria in 26.7% of transfected cells (**Supplementary Fig. 7a, b**). We also observed that mCherry and mCherry-18TM was not recruited to mitochondria in almost all transfected cells (**Supplementary Fig. 7a**, top and bottom panels, **b**). Then we tried addition of stress agents such as oxidative stress (H_2O_2) and hyperosmotic stress (high concentration of sucrose or NaCl) to the same culture condition. Two hours after H_2O_2 treatment (final concentration: 100 and 500 μM), YFP-FL showed co-localization with CFP-MoA, but co-localization with CFP was not detected in YFP-alone control cells at all (**Supplementary Fig. 7c, d**). In contrast, YFP-18TM was not co-localized with mitochondrial CFP signals. Results showing a similar trend were obtained in hyperosmotic stress condition (**Supplementary Fig. 7e-h**). Though sucrose addition at 500 mM induced mitochondrial translocation of 18TM only in 12.6% of transfected cells, the other hyperosmotic condition hardly elicited it (**Supplementary Fig. 7f, h**). In summary, FL was translocated to mitochondria upon oxidative stress or hyperosmolarity challenge, but 18TM was largely not. These stress-induced recruitments were observed in cells transfected with LD mutant of FL, but not in ones transfected with mCherry or mCherry-18TM-LD (**Supplementary Fig. 8a-h**). These results indicate that oxidative and hyperosmotic stress in addition to direct membrane damage can induce relocation of PLAAT3 onto mitochondria, which was independent of PLA activity (**Supplementary Fig. 7i**), while18TM does not show spontaneous or stress-induced relocalization.

Supplementary Note 2

Step-wise development of 18TM-induced mitochondrial deformation

To address fine morphological changes following 18TM recruitment, we analyzed serial fluorescent images. We used COS-7 cells co-transfected with mCherry-FKBP-18TM and CFP-FRB-MoA. At first, blebbing occurred in initially tubular mitochondria 2-7 minutes after rapamycin treatment (**Fig. 2a-c**, ROI-1, ROI: region of interest). Then, blebs were swollen and exhibited pearling-like deformation about 9 minutes after rapamycin treatment. Subsequently, swelling went on and each bleb was finally torn off, meaning that mitochondria was fully fragmented and took round shape (**Fig. 2a-c**, ROI-2). A part of round forms of mitochondria shrank and about 36 minutes after rapamycin addition, C-shaped mitochondria were seen (**Fig. 2a-c**, ROI-3).

Supplementary Note 3

Effect of 18TM recruitment on mitochondrial phospholipid composition

To assess lipid modification of the mitochondria membrane by the recruited 18TM, we employed fluorescently-labeled phosphatidylserine (PS) where an NBD fluorescent dye is covalently linked to PS at its Sn2 position. This NBD-PS molecule localizes to organelles such as Golgi and mitochondria in cultured cells[2,3]. We assumed that cleavage of the Sn2 fatty acid by a phospholipase leads to its release from the mitochondria membrane. Since many types of cells and tissues release intracellular free fatty acids into the extracellular space[4–7], we further expected that the NBD-labeled free fatty acids eventually released from cells, resulting in overall decrease of the cellular NBD fluorescence. HeLa cells expressing YFP-FRB-MoA and mCherry-FKBP-18TM were incubated with NBD-PS for 30 minutes, and then subjected to operation of the CID-mediated 18TM recruitment to the mitochondria. After 90 minutes of rapamycin addition, we measured NBD fluorescence intensity in cells with successful deformation of mitochondria (**Supplementary Fig. 10a**). The measured intensity value was normalized to the NBD fluorescence intensity of its nearest neighbor cell with no 18TM expression (**Supplementary Fig. 10b**), which was calculated to be 0.71 ± 0.04 (orange bar) and was significantly lower than the corresponding value before rapamycin addition (1.09 ± 0.03, blue bar, p = 0.0012). As a control, we performed the same experiment with the catalytically-inactive 18TM mutant (C113S). The normalized NBD intensity before and after rapamycin treatment was calculated to be 1.11 ± 0.02 (grey bar) and 1.08 ± 0.04 (yellow bar), respectively. This result supports the notion of mitochondrially recruited 18TM exerting its phospholipase activity to remodel membrane lipids.

Supplementary Figures

Supplementary Figure 1

a

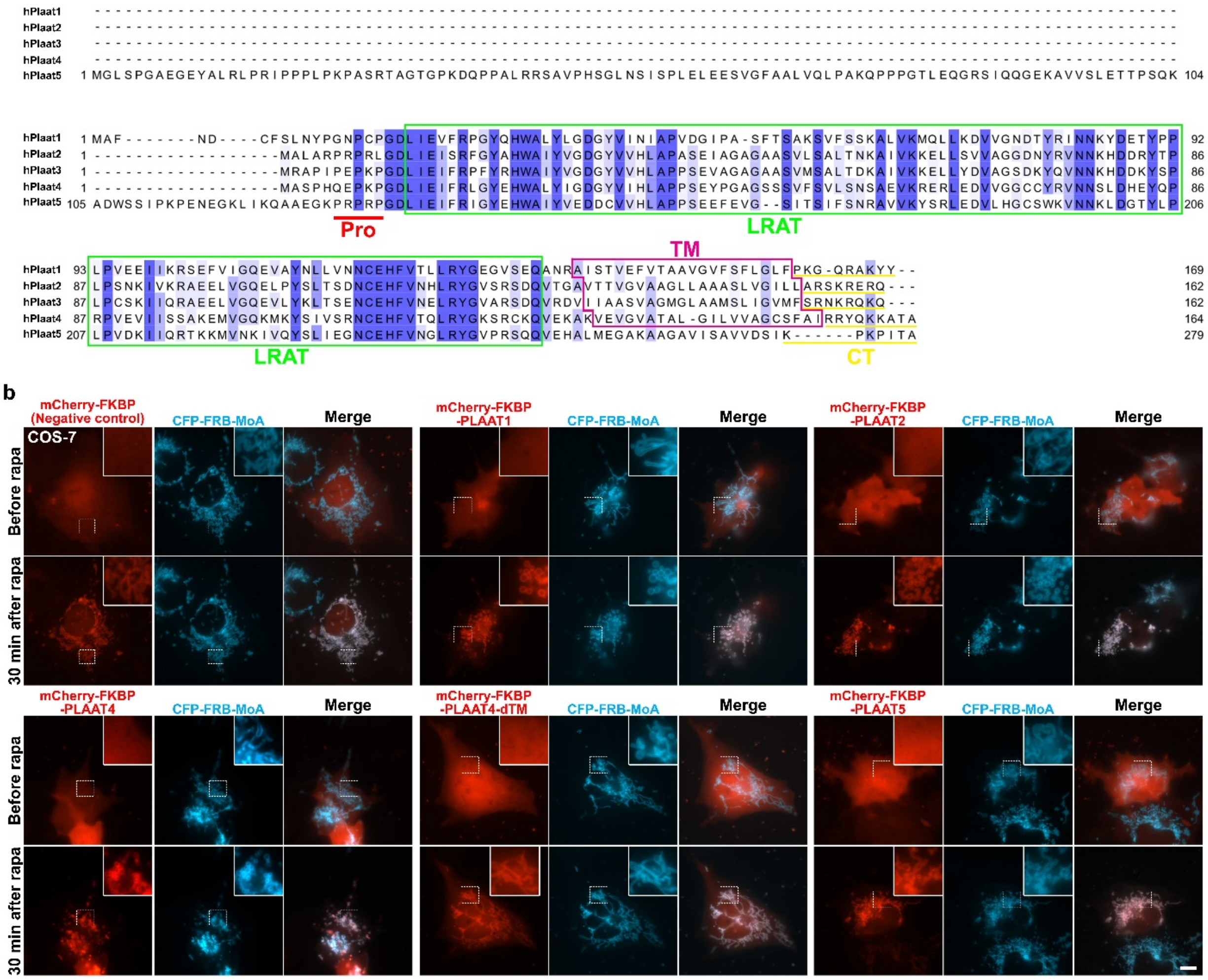

Supplementary Figure 1.

a Sequence alignment of amino acids of human PLAAT family proteins. PLAAT1: NP_065119, PLAAT2: NP_060348, PLAAT3: NP_009000, PLAAT4: NP_004576, and PLAAT5: NP_ 473449. Pro: Proline-rich domain, LRAT: lecithin-retinol acyltransferase, TM: putative transmembrane domain, CT: c-terminal domain. LRAT and TM domains were assigned based on information of UniProtKB. Sequences were aligned by Clustal Omega. **b** Fluorescent images of cells expressing mCherry-FKBP-PLAAT1, PLAAT2, PLAAT4, PLAAT4-dTM and PLAAT5 along with CFP-FRB-MoA (mitochondria outer membrane anchor protein) before and after rapamycin treatment. mCherry-FKBP was used as a negative control. Insets indicate high magnification images. The experiment was repeated at least two times. Scale bar = 10 µm. Rapa: rapamycin.

Supplementary Figure 2

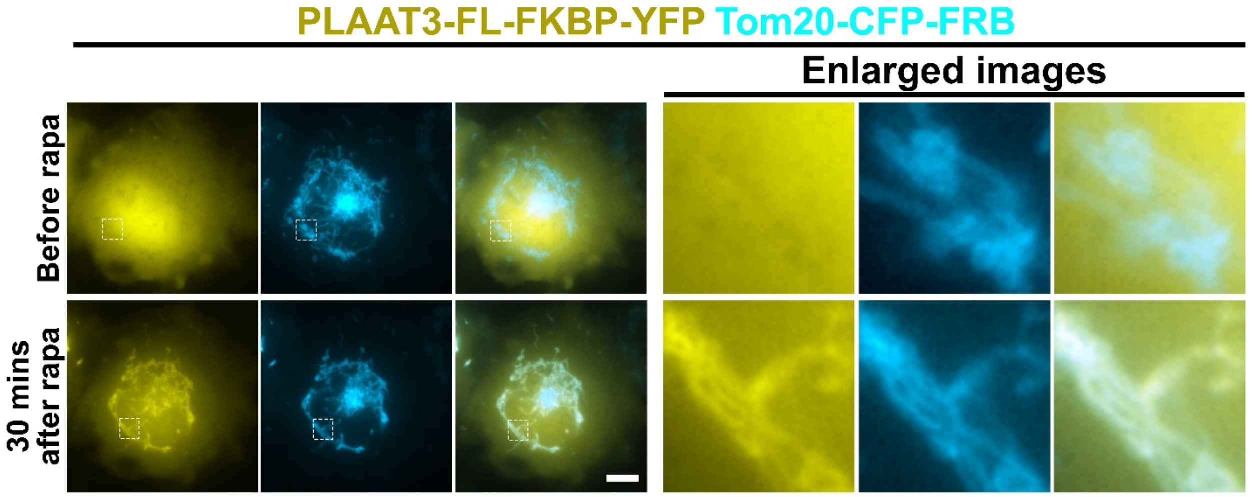

Supplementary Figure 2. C-terminal fusion of a dimerizing unit to PLAAT3-FL abrogated mitochondrial deformation activity

A full-length PLAAT3 was fused C-terminally with FKBP-YFP. A CID deformation assay was conducted in COS-7 cells, but mitochondria deformation was not induced after rapamycin treatment for 30 minutes. Areas marked with dashed white boxes were enlarged on the right side. The experiment was performed once. Scale bar = 10 µm. Rapa: rapamycin.

Supplementary Figure 3

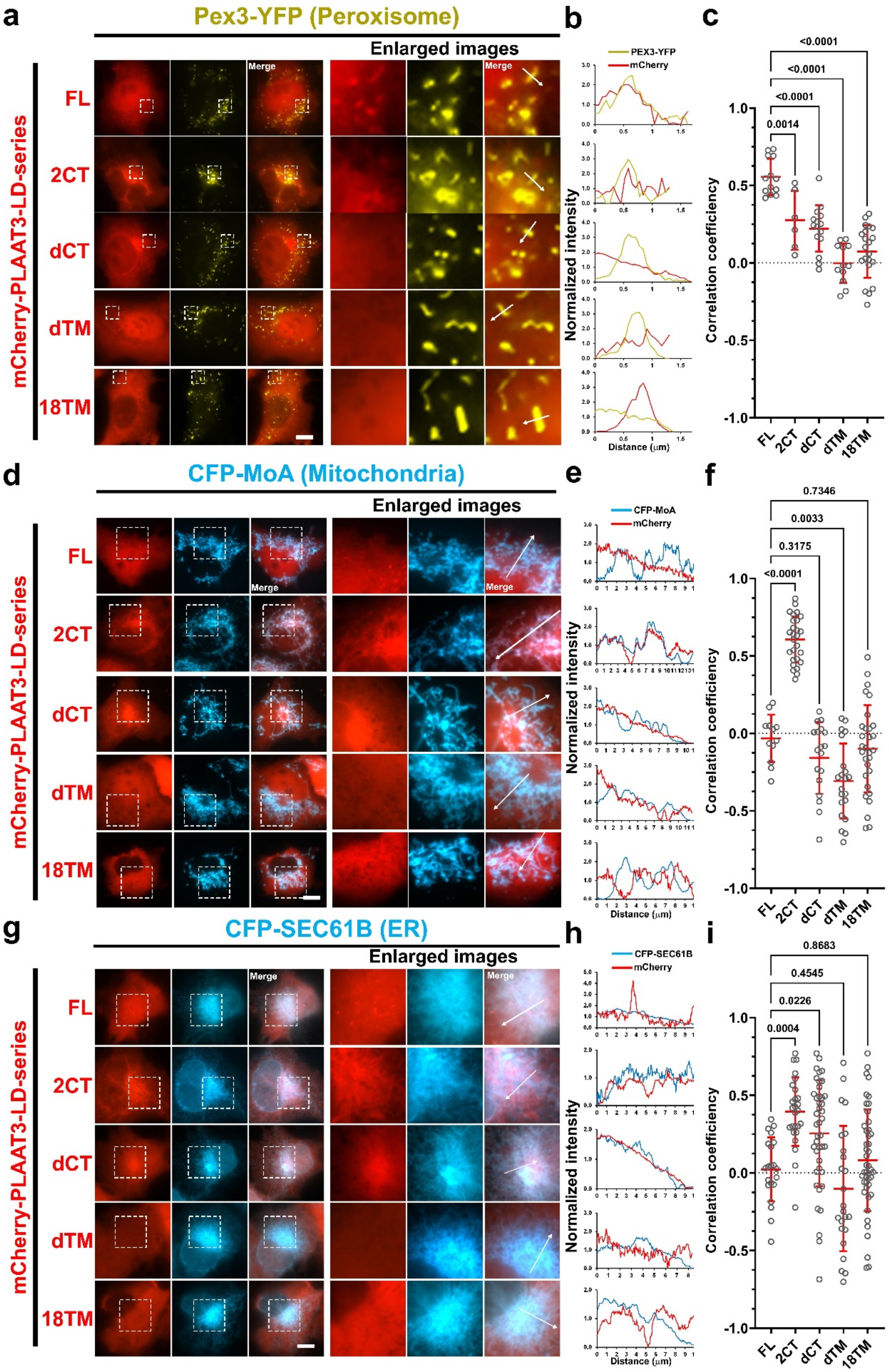

Supplementary Figure 3. Subcellular localization of PLAAT3 truncation mutants

Colocalization analyses of the PLAAT3 truncation mutants, FL, dCT, dTM, 2CT, and 18TM, with organelles such as peroxisomes, ER and mitochondria. **a-c** Based on fluorescence images shown in **a**, their line-scan trances were shown (**b**) and correlation coefficients were measured (**c**). **d-©** The analysis was repeated for the same set of PLAAT3 truncation mutants but now with ER using CFP-SEC61B (**d-f**) or with mitochondria using CFP-MoA (**g-i**). For the line-scan analysis, fluorescent intensities were analyzed along white arrows in the corresponding images, and normalized by their average intensity. Cells were analyzed one day after transfection. The result of these subcellular localization analysis is summarized in Fig. 1c. Scale bar = 10 µm. Regions of interest (ROIs) were manually selected (13, 6, 15, 12 and 21 ROIs for **c** from left to right, 12, 27, 18, 21 and 30 ROIs for **f** from left to right, 21, 26, 42, 24, and 45 ROIs for **i** from left to right, ROIs were derived from more than three biologically independent cells). Correlation coefficients were calculated with MetaMorph imaging software. Statistical significance was determined by one-way ANOVA with Dunnett's multiple comparison and p values were indicated on graphs (**c**, **f**, and **i**). Error bars indicate means ± s.d.

Supplementary Figure 4

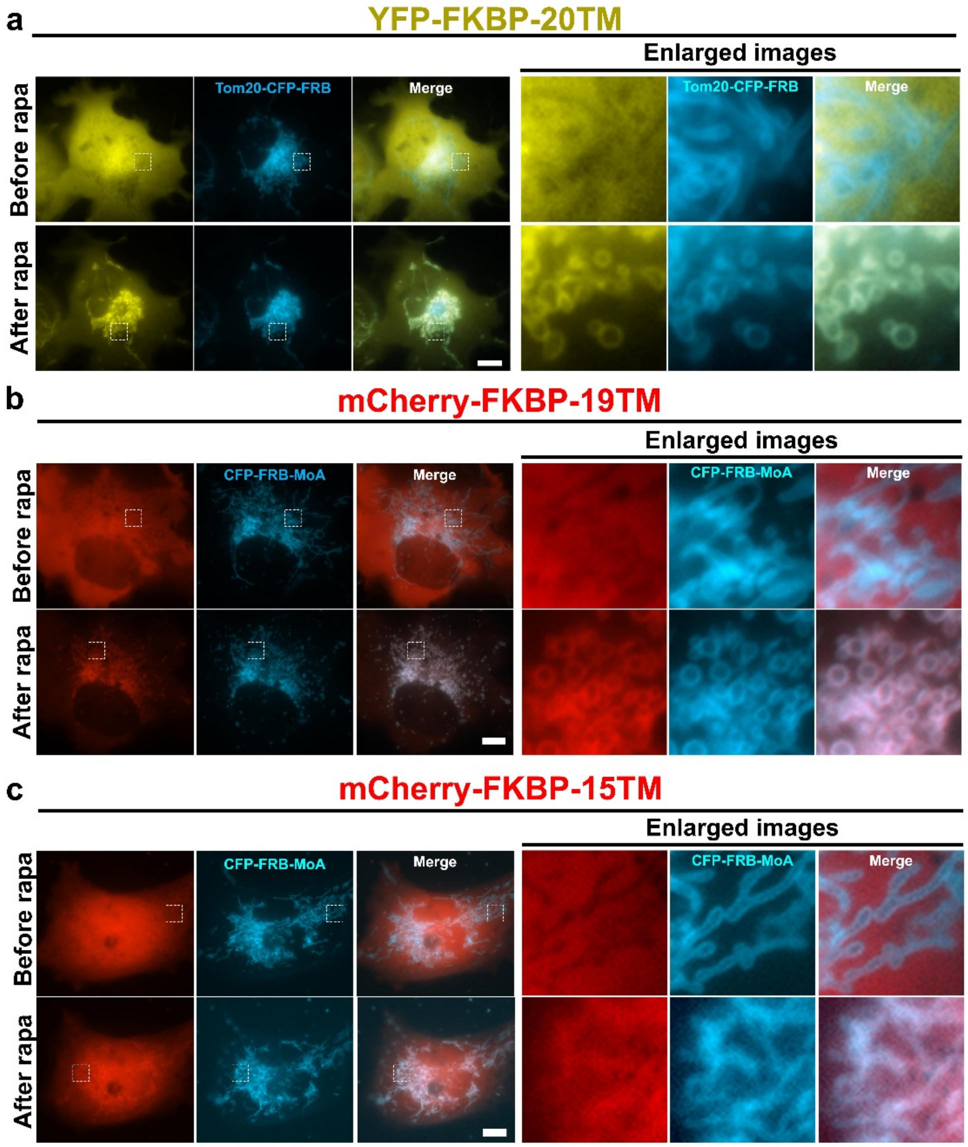

Supplementary Figure 4. Subcellular localization and mitochondrial deformation activity of PLAAT3-15TM, 19TM and 20TM

a-c Representative images of fluorescence of COS-7 cells expressing 15TM (**a**), 19TM (**b**) and 20TM (**c**) before and after rapamycin treatment. These truncation mutants were localized in cytosol and did not show apparent mislocalization of any membrane organelle (**a-c**, upper panels). Activity of inducible deformation of mitochondria was seen in 19TM- and 20TM-expressing cells, but not in 15TM-expressing cells (**a-c**, lower panels). Areas marked with dashed white boxes were enlarged on the right side. The experiment was performed once. Scale bar = 10 μm. Rapa: rapamycin.

Supplementary Figure 5

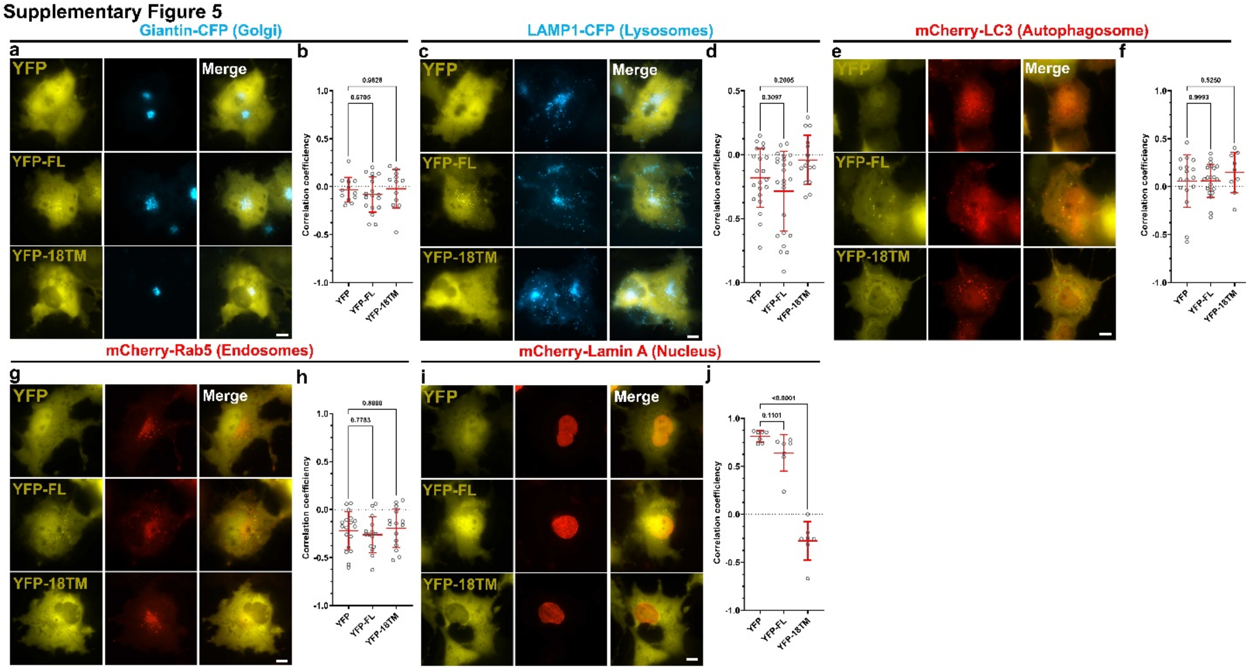

Supplementary Figure 5. Localization of PLAAT3-FL and -18TM on other membrane-bound organelles

a-**j** Subcellular localization of FL and 18TM on Golgi apparatus (Giantin-CFP) (**a, b**), lysosomes (LAMP1-CFP) (**c, d**), autophagosomes (mCherry-LC3) (**e, f**), endosomes (mCherry-Rab5) (**g, h**), and nucleus (mCherry-Lamin A) (**i, j**). To detect autophagosomes, cells were treated with chloroquine (final concentration: 100 μM) for one overnight. YFP-alone sample reflects cytosolic localization pattern. No correlation between each organelle marker signal and signal from FL or 18TM. Regions of interest (ROIs) were manually selected (12, 18 and 12 ROIs for **b** from left to right, 21, 24 and 15 ROIs for **d** from left to right, 17, 21 and 9 ROIs for **f** from left to right, 18, 15 and 15 ROIs for **h** from left to right, 7, 7 and 7 ROIs for **j** from left to right, ROIs were derived from more than three biologically independent cells). Correlation coefficiencies were calculated with MetaMorph imaging software. Statistical significance was determined by one-way ANOVA with Dunnett's multiple comparison and p values were indicated on graphs (**b, d, f, h** and **j**). Error bars indicate means ± s.d. Scale bar = 10 μm.

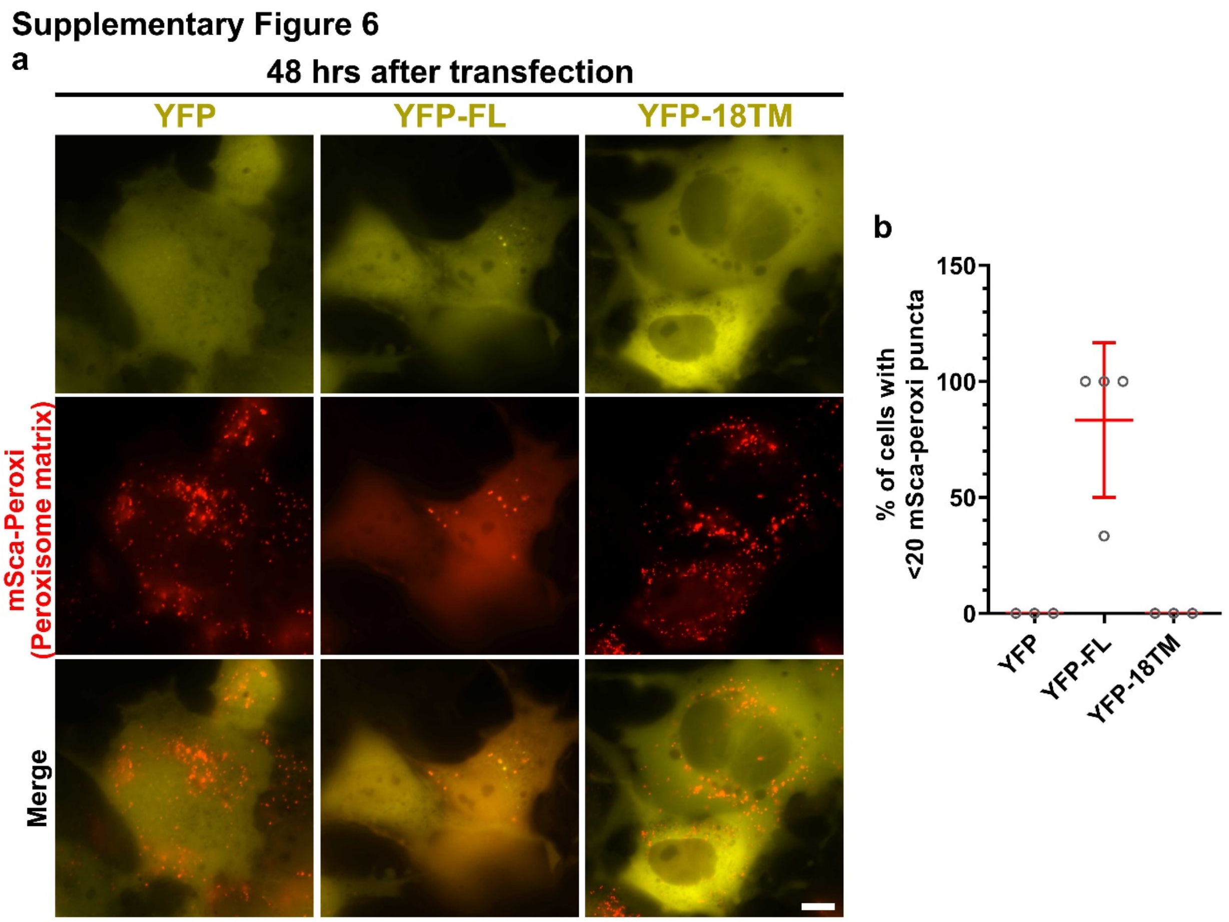

Supplementary Figure 6. An effect of PLAAT3-18TM expression on peroxisomes

Effect of FL and 18TM expression on peroxisomal number. Cells were transfected with plasmids expressing YFP (negative control), YFP-FL, and YFP-18TM and imaged at 48 hrs after transfection. FL expression reduced dot signals from mSca-peroxi, but 18TM expression did not. Cells with <20 mSca-Peroxi+ puncta were manually counted in 3, 4 and 3 fields of view from left to right (n = 3 or 4 biologically independent cells). Statistical significance was determined by one-way ANOVA with Dunnett's multiple comparison and *p* values were indicated on graphs (**b**). Error bars indicate means ± s.d. Scale bar = 10 µm.

Supplementary Figure 7

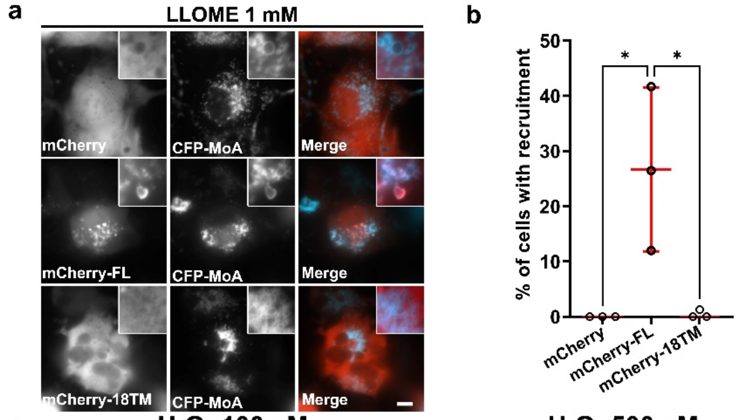

i

	Spontaneous recruit	Stress-induced recruit
FL	+	+
FL-LD	+	+
18TM	-	-
18TM-LD	-	-

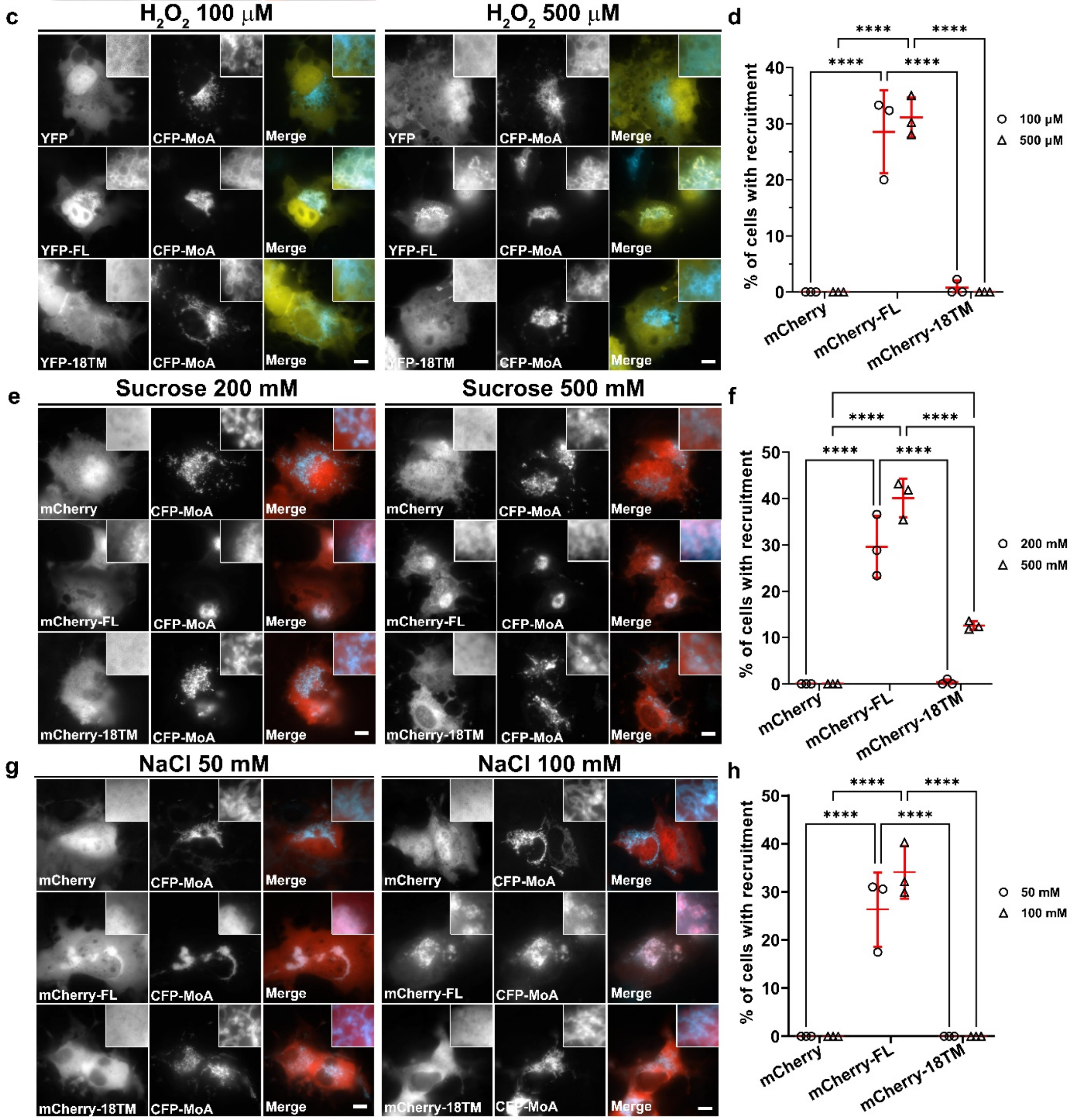

Supplementary Figure 7. Relocation of PLAAT3-FL and -18TM upon oxidative or hyperosmotic stress

a Observation of relocation of FL and 18TM 1 hr after treatment with membrane damage agent (1mM LLOME). **b** Quantification of **a**. n = 405, 305 and 361 cells from left to right; analyzed from three individual experiments. Fluorescent images of cells treated with oxidative stress (100 μM or 500 μM of H_2O_2) or hyperosmotic stress (200 mM or 500 mM of Sucrose, 50 mM or 100 mM of NaCl) for 2 hrs. **d**, **f**, **h** Quantification of **c**, **e** and **g**. n = 222, 240, 213, 255, 192 and 252 cells from left to right; analyzed from three individual experiments (**d**). n = 373, 379, 273, 265, 277 and 216 cells from left to right; analyzed from three individual experiments (**f**). n = 297, 264, 206, 273, 229 and 234 cells from left to right; analyzed from three individual experiments (**h**). Percentage of cells with recruitment of mCherry, mCherry-FL, and mCherry-18TM on mitochondria was calculated. **i** Summary of spontaneous recruit and stress-induced recruit of FL, FL-LD, 18TM and 18TM-LD. Data from Fig. 1e and data from Supplementary Fig. 6 were integrated to **i** for summary. Insets indicate high magnification images. Error bars indicate means ± s.d.. Statistical significance was determined by one-way ANOVA with Dunnett's multiple comparison (**b**) and two-way ANOVA with Tukey's multiple comparison (**d**, **f** and **h**). and *p* values were indicated on graphs (**b**, **d**, **f**, and **h**). Cells with or without YFP accumulation on mitochondria were manually counted. Scale bar = 10 μm.

Supplementary Figure 8

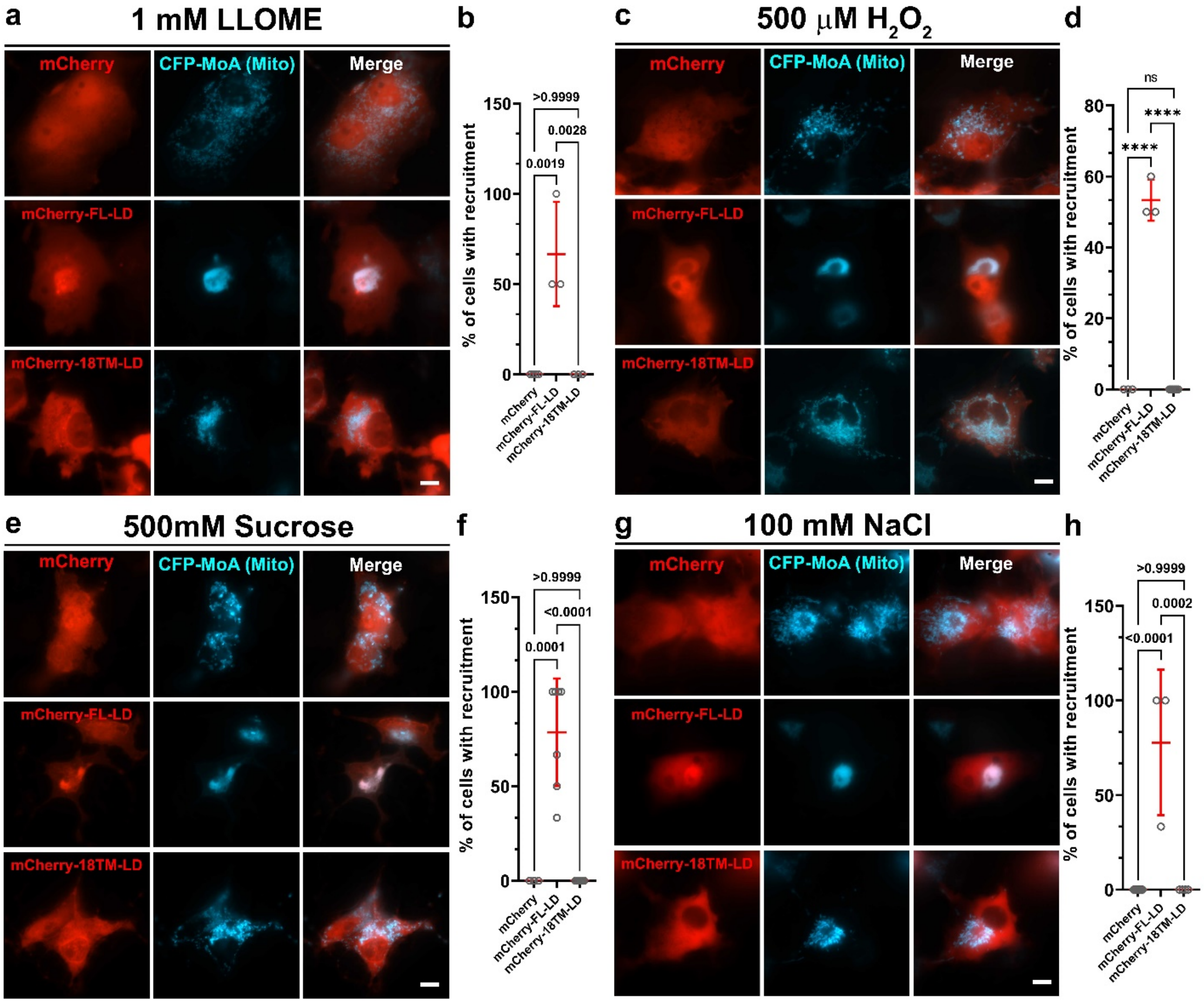

Supplementary Figure 8. Stress-induced relocation was independent on PLA activity

Based on fluorescence images of COS-7 cells co-expressing CFP-MoA (mitochondria marker) and either mCherry, mCherry-FL-LD or mCherry-18TM-LD, which were treated with 1 mM LLOME (**a**), 500 μM H_2O_2 (**c**), 500mM sucrose (**e**), or 100mM NaCl (**g**). Cells with or without mCherry accumulation on mitochondria were then manually counted for each treatment condition and plotted [**b, d, f, h,** n = 4, 3, 3 (**b**), n = 3, 3, 6 (**d**) n = 3, 7, 6 (**f**) n = 7, 3, 4 (**h**), biologically independent cells). Statistical significance was determined by one-way ANOVA with Dunnett's multiple comparison and *p* values were indicated on graphs. Error bars indicate means ± s.d. Scale bar = 10 μm.

Supplementary Figure 9

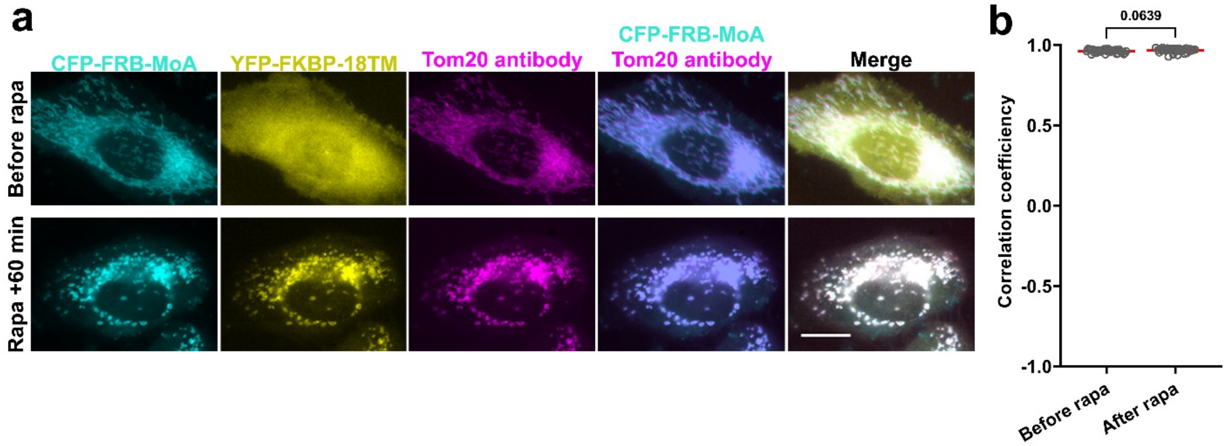

Supplementary Figure 9. Colocalization between CFP-FRB-MoA and endogenous Tom20 in cells with or without 18TM recruitment.

a Cells co-expressing CFP-FRB-MoA and YFP-FKBP-TM18 were incubated with or without rapamycin for 60 minutes prior to staining with an antibody against endogenous Tom20. Fluorescence images of CFP-FRB-MoA (cyan), YFP-FKBP-TM18 (yellow) and Alexa647 (magenta) are shown along with double merge (CFP-FRB-MoA and YFP-FKBP-TM18) and triple merge (**a**). **b** The colocalization coefficient values were calculated from 35 and 33 biologically independent cells for samples before and after rapamycin addition from three independent experiments [(rapamycin-untreated (0.961 ± 0.001) and rapamycin-treated (0.966 ± 0.002)). Statistical significance was determined by paired two-tailed *t*-test and *p* values were indicated on graphs (**b**). Error bars indicate means ± s.d.. Scale bar = 20 μm.

Supplementary Figure 10

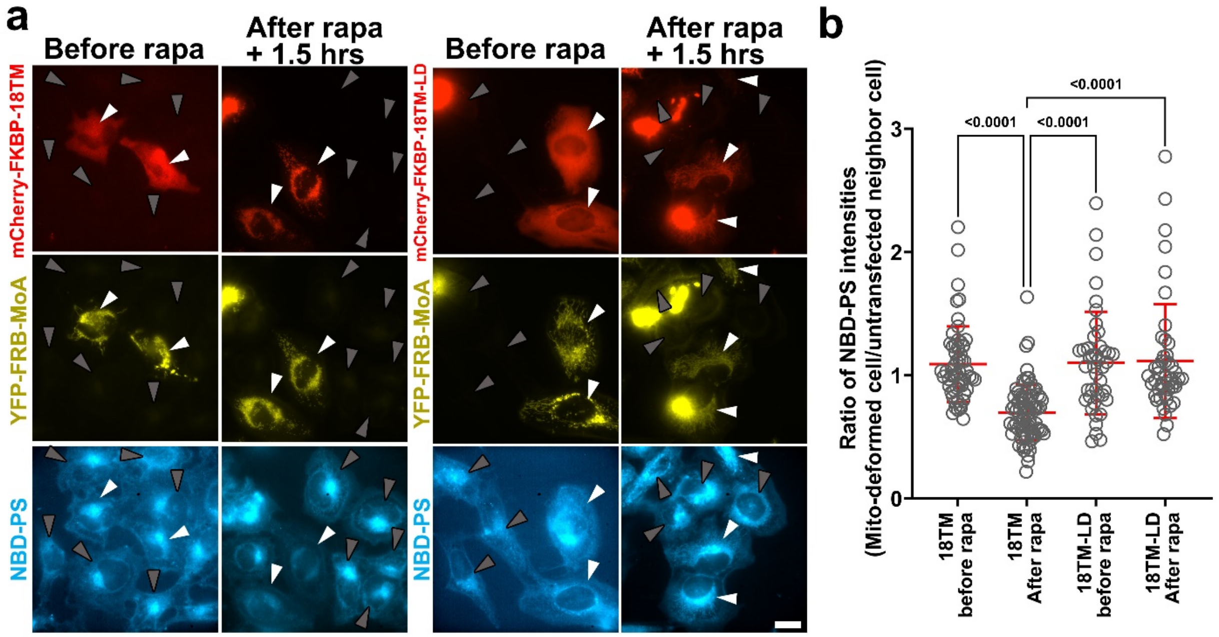

Supplementary Figure 10. Measurement of membrane phospholipids before and after the 18TM-mediated mitochondria deformation.

a Cells co-expressing YFP-FRB-MoA and either mCherry-FKBP-18TM or mCherry-FKBP-18TM-LD (C113S) were incubated with NBD-PS for 30 minutes prior to rapamycin addition. Fluorescence images of NBD-PS and mCherry-FKBP fusion proteins were captured in each of these four conditions before and 90 minutes after rapamycin addition. White arrowheads indicate cells transfected with YFP-FRB-MoA and mCherry-FKBP-18TM (or -18TM-LD). Gray arrowheads indicate untransfected cells. Scale bar = 20 μm. Rapa: rapamycin. **b** Fluorescence intensity of NBD-PS was measured in each of the above four conditions and normalized to the intensity of its nearest neighbor cell with no expression of 18TM or 18TM-LD. A calculated value for 18TM was 0.71 ± 0.04 (orange bar) which was significantly lower than the corresponding value before rapamycin addition (1.09 ± 0.03, blue bar, p = 0.0012). Calculated values for 18TM-LD with or without rapamycin treatment were 1.11 ± 0.02 (grey bar) and 1.08 ± 0.04 (yellow bar), respectively. The MetaMorph imaging Software was used in this analysis. The plots reflect analysis of 57, 78, 43 and 46 biologically independent cells per respective conditions from more than three different set of experiments. Error bars indicate means ± s.d.. Statistical significance was determined by one-way ANOVA with Dunnett's multiple comparison and p values were indicated on graphs.

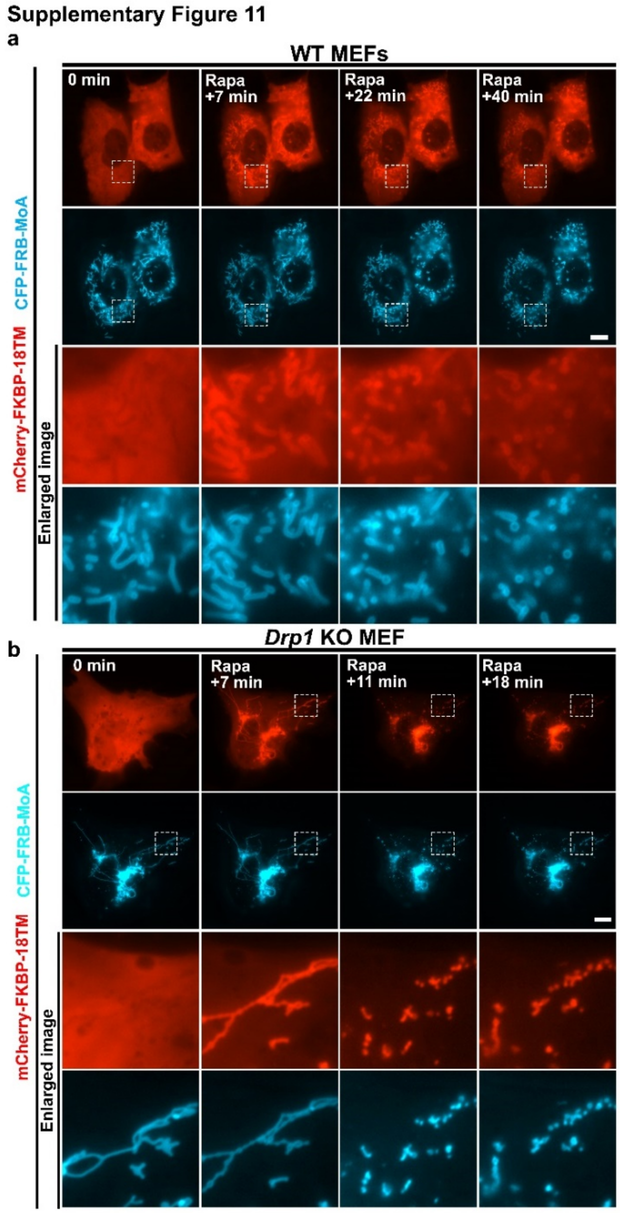

Supplementary Figure 11. PLAAT3-18TM-mediated fragmentation was independent of mitochondrial fission exerted by DRP1

a, **b** Representative images of WT (**a**) or *Drp1* KO (**b**) MEFs expressing mCherry-FKBP-18TM and CFP-FRB-MoA before and after rapamycin treatment. In *Drp1* KO MEFs, only elongated forms of mitochondria were seen. In both cell types, mitochondrial deformation by 18TM was induced. The experiment was performed once, but the same results were reproduced in at least three different cells. Scale bar = 10 μm. Rapa: rapamycin.

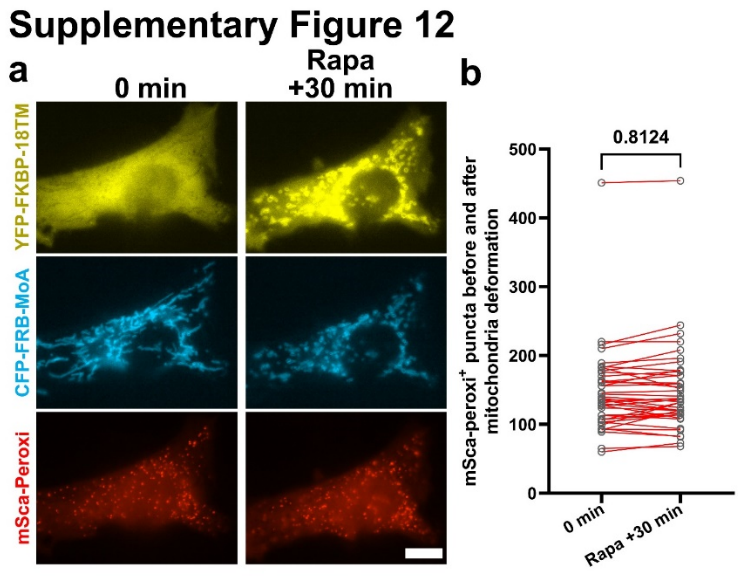

Supplementary Figure 12. Specific action of 18TM recruitment on mitochondria

a Representative images of cells expressing CFP-FRB-MoA (blue), YFP-FKBP-18TM (yellow) and mSca-Peroxi (red) at 0 and 30 minutes after rapamycin addition. **b** Quantification of number of peroxisomes (bottom panels) in individual cells from three separate experiments did not lead to detectable changes between the two time points (145 ± 11 at 0 min vs. 148 ± 13 at 30 min, total of 41 biologically independent cells). Statistical significance was determined by paired two-tailed *t*-test and *p* values were indicated on the graph. Error bars indicate means ± s.d.

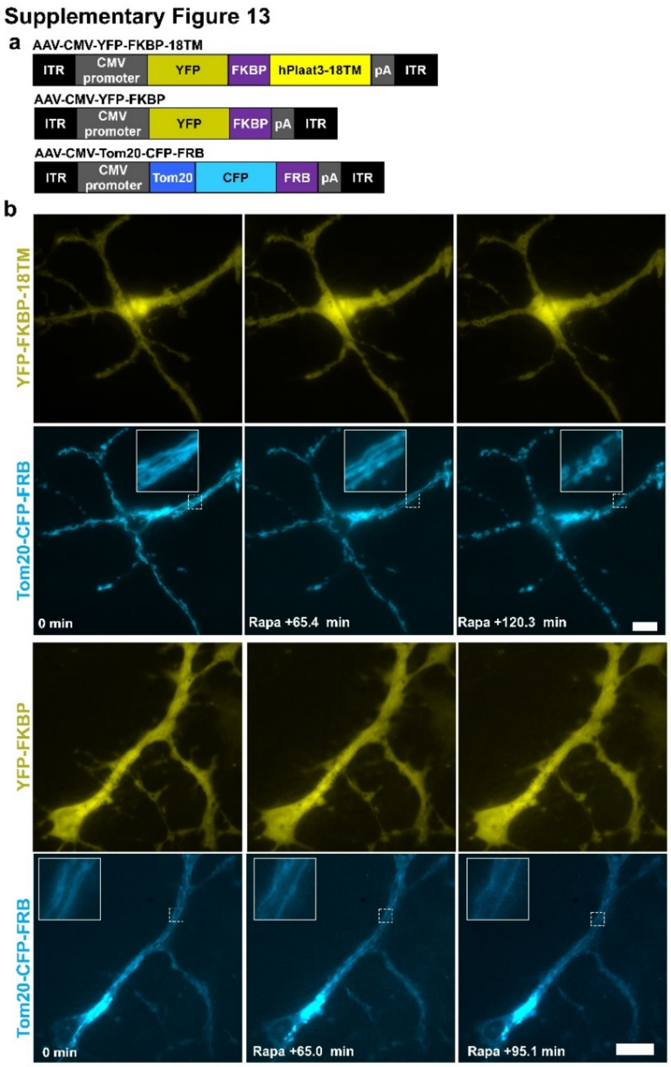

Supplementary Figure 13. Induced mitochondrial deformation following AAV-mediated delivery of 18TM

a Schematic diagram of AAV transgenes. Three types of AAVs were generated and infected to mouse primary hippocampal neurons. The fist type of AAV expresses YFP-FKBP-18TM, second expresses YFP-FKBP-alone and third expresses Tom20-CFP-FRB (mitochondrial outer membrane anchor). Expression of all genes was driven by CMV promoter. AAV-CMV-YFP-FKBP was used as a negative control. ITR: inverted terminal repeat. Viruses were added to neurons at 6 DIV at MOI = 40,000. Two days after infection, cells were analyzed. **b** Representative images of mitochondrial morphology in neurons. Mitochondrial deformation after 18TM translocation was induced in YFP-FKBP-18TM-expressing neurons. The experiment was repeated twice. Scale bar = 10 μm. Rapa: rapamycin.

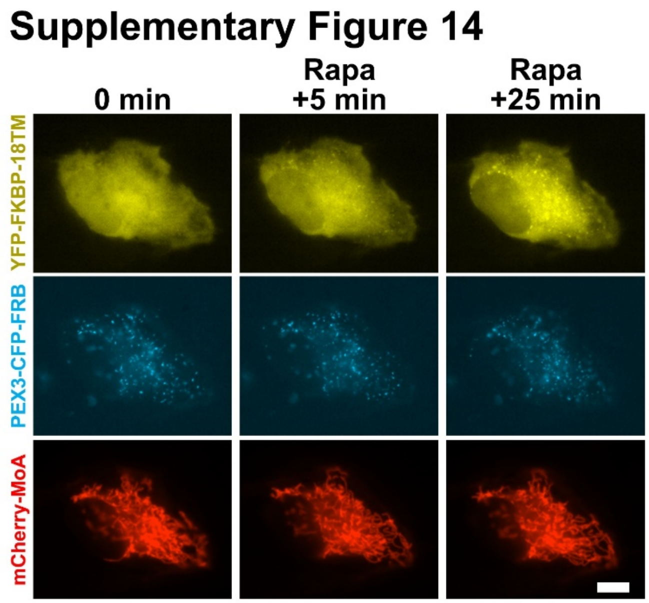

Supplementary Figure 14. Specific action of 18TM recruitment on peroxisomes

Representative images of cells expressing PEX3-CFP-FRB (blue), YFP-FKBP-18TM (yellow) and mCherry-MoA (red) are shown at 0, 5 and 25 minutes after rapamycin addition. While we observed rapamycin-induced 18TM accumulation at the peroxisomes (middle panels), there was no detectable mitochondria deformation, which was the case in all 23 cells we inspected in three separate experiments. Scale bar = 10 µm. Rapa: rapamycin.

Supplementary References

1. Morishita, H. *et al.* Organelle degradation in the lens by PLAAT phospholipases. *Nature* **592**, 634–638 (2021).
2. Kobayashi, T. & Arakawa, Y. Transport of exogenous fluorescent phosphatidylserine analogue to the Golgi apparatus in cultured fibroblasts. *J. Cell Biol.* **113**, 235–244 (1991).
3. Komatsu, T. *et al.* Organelle-specific, rapid induction of molecular activities and membrane tethering. *Nat. Methods* **7**, 206–208 (2010).
4. Evans, J. R. CELLULAR TRANSPORT OF LONG CHAIN FATTY ACIDS. *Can. J. Biochem.* **42**, 955–969 (1964).
5. Porte, D. & Entenman, C. FATTY ACID METABOLISM IN SEGMENTS OF RAT INTESTINE. *Am. J. Physiol.* **208**, 607–614 (1965).
6. Spector, A. A. & Steinberg, D. Release of free fatty acids from Ehrlich ascites tumor cells. *J. Lipid Res.* **7**, 649–656 (1966).
7. Figard, P. H., Hejlik, D. P., Kaduce, T. L., Stoll, L. L. & Spector, A. A. Free fatty acid release from endothelial cells. *J. Lipid Res.* **27**, 771–780 (1986).
