# Supplementary material for: Defunctionalizing intracellular organelles such as mitochondria and peroxisomes with engineered phospholipase A/acyltransferases
Source: Nat Commun. 2022 Jul 29;13:4413. doi: 10.1038/s41467-022-31946-5 (PMC9338259; doi:10.1038/s41467-022-31946-5)
Supplement: Supplementary file 3 — Description of Additional Supplementary Files [file 41467_2022_31946_MOESM3_ESM.pdf]

**Title: Supplementary Movies 1.**

**Description:** Inducible mitochondrial deformation using PLAAT3-FL. COS-7 cells were transfected with mCherry-FKBP-PLAAT3-FL and CFP-FRB-MoA. The upper panels of **Fig. 1** were extracted as representative images based on this movie. Rapamycin was added at t = 5 mins. Scale bar = 10 μm.

**Title: Supplementary Movies 2.**

**Description:** A lipase dead mutant of PLAAT3 did not induce mitochondrial deformation. COS-7 cells were transfected with mCherry-FKBP-PLAAT3-FL-LD and CFP-FRB-MoA. The lower panels of **Fig. 1** were extracted as representative images based on this movie. Rapamycin was added at t = 5 mins. Scale bar = 10 μm.

**Title: Supplementary Movies 3.**

**Description:** More detailed morphological changes of mitochondria upon a CID-18TM recruitment. COS-7 cells were transfected with mCherry-FKBP-18TM and CFP-FRB-MoA. The images of **Fig. 2a** were extracted as representative images based on this movie. Rapamycin was added at t = 1 min. Scale bar = 10 μm.

**Title: Supplementary Movies 4.**

**Description:** Loss of membrane potential following the 18TM-medtiated deformation. COS-7 cells were transfected with YFP-FKBP-18TM and CFP-FRB-MoA. The images of **Fig. 3a** were extracted as representative images based on this movie. Rapamycin was added at t = 9 mins. Scale bar = 10 μm.

**Title: Supplementary Movies 5.**

**Description:** Su9-CFP leakage following the 18TM-mediated deformation. COS-7 cells were transfected with YFP-FKBP-18TM, Su9-CFP, and mCherry-FRB-MoA. The images of **Fig. 4a** were extracted as representative images based on this movie. This movie shows only CFP (blue) and mCherry (red) channels. Scale bar = 10 μm.

**Title: Supplementary Movies 6.**

**Description:** Su9-CFP did not leak out of mitochondria in 18TM-LD-expressing cells. COS-7 cells were transfected with YFP-FKBP-18TM-LD, Su9-CFP, and mCherry-FRB-MoA. The images of **Fig. 6c** were extracted as representative images based on this movie. This movie shows only CFP (blue) and mCherry (red) channels. Scale bar = 10 μm.

**Title: Supplementary Movies 7.**

**Description:** Dissipation of matrix-resident proteins of peroxisomes following the 18TM recruitment. HeLa cells were transfected with YFP-FKBP-18TM, PEX3-CFP-FRB, and mSca-Peroxi. The images of **Fig. 6a** were extracted as representative images based on this movie. Rapamycin was added at t = 1 min. Scale bar = 10 μm.

**Title: Supplementary Movies 8.**

**Description:** mSca-Peroxi proteins were not dissipated following the 18TM-LD recruitment. HeLa cells were transfected with YFP-FKBP-18TM-LD, PEX3-CFP-FRB, and mSca-peroxi. The images of **Fig. 6b** were extracted as representative images based on this movie. Rapamycin was added at t = 1 min. Scale bar = 10 μm.
